# A novel optimization rainfall coupling model based on stepwise decomposition technique

**DOI:** 10.1038/s41598-024-66663-0

**Published:** 2024-07-06

**Authors:** Zhiwen Zheng, Xianqi Zhang, Qiuwen Yin, Fang Liu, He Ren, Ruichao Zhao

**Affiliations:** 1 Water Conservancy College, North China University of Water Resources and Electric Power, Zhengzhou, 450046 China; 2 Collaborative Innovation Center of Water Resources Efficient Utilization and Protection Engineering, Zhengzhou, 450046 China; 3 Kunming Engineering Corporation Limited, Kunming, 650051 China

**Keywords:** Stepwise decomposition technique, Variational modal decomposition (VMD), African vulture optimization algorithm (AVOA), North China plain, Atmospheric science, Hydrology

## Abstract

Traditional decomposition integration models decompose the original sequence into subsequences, which are then proportionally divided into training and testing periods for modeling. Decomposition may cause data aliasing, then the decomposed training period may contain part of the test period data. A more effective method of sample construction is sought in order to accurately validate the model prediction accuracy. Semi-stepwise decomposition (SSD), full stepwise decomposition (FSD), single model semi-stepwise decomposition (SMSSD), and single model full stepwise decomposition (SMFSD) techniques were used to create the samples. This study integrates Variational Mode Decomposition (VMD), African Vulture Optimization Algorithm (AVOA), and Least Squares Support Vector Machine (LSSVM) to construct a coupled rainfall prediction model. The influence of different VMD parameters α is examined, and the most suitable stepwise decomposition machine learning coupled model algorithm for various stations in the North China Plain is selected. The results reveal that SMFSD is relatively the most suitable tool for monthly precipitation forecasting in the North China Plain. Among the predictions for the five stations, the best overall performance is observed at Huairou Station (RMSE of 18.37 mm, NSE of 0.86, MRE of 107.2%) and Jingxian Station (RMSE of 24.74 mm, NSE of 0.86, MRE of 51.71%), while Hekou Station exhibits the poorest performance (RMSE of 25.11 mm, NSE of 0.75, MRE of 173.75%).

## Introduction

Strong and frequent rainstorms have put human security and socioeconomic well-being in grave danger in recent times. Reliable rainfall process modeling is critical to water resource management. However, a multitude of intricate and unpredictable mechanisms impact the development of precipitation^1^. Process-driven and data-driven methodologies can be used to broadly classify the models of precipitation prediction now in use. Unlike process-driven models, data-driven models are exempt from taking into account the physical mechanisms underlying runoff generation^2,3^. Rather, they only use mathematical analysis of time-series data to determine the functional relationships between the variables that are input and output, which increases tractability^4^.

With the development of artificial intelligence, especially the application of machine learning techniques, new opportunities are provided to improve rainfall prediction methods^5,6^. Because it avoids quadratic programming problems, the SVM-based LSSVM model improves computational efficiency^7,8^. But because the parameters of an LSSVM greatly affect its predictive accuracy, some researchers have added optimization methods to LSSVM in order to find the best model parameters^9,10^. Xiang et al.^11^ used the bird flocking algorithm to optimize the parameters of the LSSVM model in order to forecast short-term wind speeds. Due to its exceptional qualities, the AVOA algorithm has been applied in numerous fields. It is fast to converge, extremely adaptive, and capable of avoiding local optimum situations^12,13^.

Zhang et al.^14^ provide evidence of the special convenience of the coupled prediction model based on decomposition-reconstruction. Decomposition improves modeling stability by enabling a more effective division of big raw data sets into several subsequences with stronger periodicity^15^. Hydrological prediction models have been shown to perform better when using common decomposition models, such as the Discrete Wavelet Transform^16^, Complete Ensemble Empirical Mode Decomposition with Adaptive Noise^17,18^, and Variational Mode Decomposition^19,20^. To train the prediction model, the majority of the current research first decompose the original data in its entirety. Then, they divide the decomposed data into training and testing periods. The full decomposition approach sampling technique's prediction model was employed by Du et al.^21^ for applicability testing. The findings indicate that the sequences produced by this method contain information about the predicted data, resulting in improved prediction performance. A machine learning and VMD coupling model was presented by Seo et al.^22^ in an effort to improve the daily rainfall-runoff modeling's accuracy. The phenomenon of data leakage between the training and testing stages, however, cannot be completely mitigated by this sampling strategy^23^. In order to assess the efficacy of the original data in predicting monthly runoff at five typical Poyang Lake stations, He et al.^20^ broke down the data using various sampling approaches. The findings show that stepwise decomposition sampling methods combined with VMD can greatly increase the model's efficiency and accuracy.

Currently, most researchers are committed to exploring new coupled machine learning models to improve the accuracy of rainfall prediction. There are fewer studies on improving rainfall prediction accuracy by comparing different sampling techniques. In this paper, a new coupled model is proposed by combining the VMD decomposition technique with LSSVM and AVOA, aiming to better deal with complex and variable rainfall processes and improve the accuracy and reliability of rainfall prediction. By comparing four stepwise decomposition sampling techniques applied to five typical stations in the North China Plain, the most suitable sampling technique for rainfall prediction in the North China Plain is identified, which provides new methods and ideas for regional rainfall prediction.

## Models and methods

### African vulture optimisation algorithm (AVOA)

The AVOA algorithm starts by assuming that there are N vultures in the search space, and it first employs a grouping strategy to enhance population diversity, i.e., the two vultures with the first (optimal) and second (suboptimal) fitness values are grouped together, and the remaining (N-2) vultures begin to search for food around the top two^24^. The following is the iterative process of the AVOA algorithm:

Stage 1: Randomly initialise the population, and then select the best or second best individual for the next stage of optimisation according to the "roulette" rule. For the ith vulture in the population, its learning object is selected according to Eq. (1):

1 $$R(i) = \left\{ {\begin{array}{*{20}l} {{\text{Optimal }}\;{\text{Vulture}}, } & {if \;rand \le L} \\ {{\text{suboptimal vulture}}, } & {else} \\ \end{array} } \right.$$

where $L$ is a user-defined parameter located between (0, 1), which contributes to the increase of population diversity when $L$ tends to 0; conversely, it accelerates population aggregation; and rand is a [0, 1] uniformly distributed random number.

Phase 2: Define the starvation rate to enable the transition between the algorithm development and exploration process. The starvation rate $F$ increases as the iterative process advances to more likely facilitate the development process.

2 $$t = h \times \left( {\sin^{w} \left( {\frac{\pi }{2} \times \frac{iter}{{\max iters}}} \right) + \cos \left( {\frac{\pi }{2} \times \frac{iter}{{\max iters}}} \right) - 1} \right)$$

3 $$F = \left( {2 \times rand + 1} \right) \times z \times \left( {1 - \frac{iter}{{\max iters}}} \right) + t$$

where, $h$, $z$ are uniformly distributed random numbers in [− 2, 2] and [− 1, 1], respectively; $w$ is a user-defined parameter that controls the probability that the algorithm enters the exploration mode in the final stage; and $iter$ is the current number of iterations as the algorithm proceeds.

Phase 3: Spatial exploration. The AVOA uses a user-defined parameter *P*_1_ to determine which exploration mode to enter, taking values between (0, 1).

4 $$P(i + 1) = \left\{ {\begin{array}{*{20}l} {R(i) - D(i) \times F,} & { if P_{1} \ge rand} \\ {R(i) - F + rand \times \left( {\left( {ub - lb} \right) \times rand_{3} + lb} \right),} & { if P_{1} < rand} \\ \end{array} } \right.$$

5 $$D(i) = \left| {XR\left( i \right) - P\left( i \right)} \right|$$

where $P(i + 1)$ is the updated position of the vulture; $X$ is a [0, 2] uniformly distributed random number.

Phase 4: Local exploitation. The AVOA initiates the exploitation phase when the absolute value of the starvation rate $\left| F \right| < 1$. Unlike the exploration phase this phase contains two types of subphases, and the initiation of the two subphases is demarcated by $\left| F \right| = 0.5$|.

Subphase 1 judgement condition: $\left| F \right| \ge 0.5$. In this phase, the position updating method mimics the characteristics of the vulture's spiral flight and is executed according to Eqs. (6) to (9):

6 $$P(i + 1) = \left\{ {\begin{array}{*{20}l} {D(i) \times \left( {F + rand} \right) - d\left( t \right),} & { if P_{2} \ge rand} \\ {R(i) - \left( {S_{1} + S_{2} } \right), } & {if P_{2} < rand} \\ \end{array} } \right.$$

7 $$d(t) = R\left( i \right) - P\left( i \right)$$

8 $$S_{1} = R\left( i \right) \times \left( {\frac{{rand_{5} \times P\left( i \right)}}{2\pi }} \right) \times \cos \left( {P\left( i \right)} \right)$$

9 $$S_{2} = R\left( i \right) \times \left( {\frac{{rand_{6} \times P\left( i \right)}}{2\pi }} \right) \times \sin \left( {P\left( i \right)} \right)$$

where the parameter $P_{2}$ takes a value between (0, 1).

Sub-stage 2 judgement condition: $\left| F \right| < 0.5$. This stage is executed according to Eqs. (10)–(12):

10 $$P(i + 1) = \left\{ {\begin{array}{*{20}l} {\frac{{A_{1} + A_{2} }}{2}, } & {if P_{3} \ge rand_{P3} } \\ {R(i) - \left| {d\left( t \right)} \right| \times F \times Levy\left( d \right),} & { if P_{3} < rand_{P3} } \\ \end{array} } \right.$$

11 $$A_{1} = {\text{Optimal \, Vulture}}\left( i \right) - \frac{{{\text{Optimal \, Vulture}}\left( i \right) \times P\left( i \right)}}{{{\text{Optimal \, Vulture}}\left( i \right) - P\left( i \right)^{2} }} \times F$$

12 $$A_{2} = {\text{suboptimal \, vulture}}\left( i \right) - \frac{{{\text{suboptimal \, vulture}}\left( i \right) \times P\left( i \right)}}{{{\text{suboptimal \, vulture}}\left( i \right) - P\left( i \right)^{2} }} \times F$$

Similarly, the parameter $P_{3}$ takes values between (0, 1).

### Variational mode decomposition (VMD)

VMD is a commonly used adaptive and fully recursive signal sequence processing method^25^, which firstly requires the number of decompositions, K, and the quadratic penalty factor, α, and then iteratively searches for the optimal centre frequency and finite bandwidth corresponding to the optimal solution of the model, which is able to adaptively match the respective intrinsic mode function (IMF) and achieve effective separation of the IMF. IMF, then iteratively search for the optimal centre frequency and finite bandwidth corresponding to the optimal solution of the model that can adaptively match each IMF and achieve effective separation of IMFs^26^.

13 $$u_{k} \left( t \right) = A_{k} \left( t \right)\cos \left[ {\varphi_{k} \left( t \right)} \right]$$

where $A_{K} (t)$ is the instantaneous amplitude function; $\varphi_{K} (t)$ is the non-decreasing instantaneous phase function. Then $\omega_{K} (t)$ = $\varphi^{\prime}_{K} (t) \ge 0$, defining $\omega_{K} (t)$ as the instantaneous frequency of $u_{K} (t)$.

### Least squares support vector machine (LSSVM)

LSSVM is an improved algorithm based on SVM^27^. The classical SVM is based on the need to minimise the structural risk minimisation principle by introducing the associated loss function and relaxation variables, and the fitting problem is transformed into solving a quadratic optimisation problem. The improvement made by the LSSVM is that the inequality constraints in this optimisation problem are converted into equality constraints, and the following optimal objective function is constructed:

14 $$\left\{ {\begin{array}{*{20}l} {\min \frac{1}{2}\left\| {\varvec{w}} \right\|^{2} + C\frac{1}{2}\sum\limits_{i = 1}^{n} {\delta_{i}^{2} } } \hfill \\ {{\text{s}}{\text{.t}}{. }Y_{i} = \left\langle {{\varvec{w}},\Phi \left( {{\varvec{X}}_{i} } \right)} \right\rangle + b + \delta_{i} ,i = 1,2, \ldots ,n} \hfill \\ \end{array} } \right.$$

where $C$ is the regularisation parameter and $\delta_{i}$ is the ith relaxation variable. By introducing the Lagrange factor $\alpha_{i}$, the Lagrange function $L$ can be written as:

15 $$L(w,b,\delta ,\varepsilon ) = \frac{1}{2}\left\| {\varvec{w}} \right\|^{2} + C\frac{1}{2}\sum\limits_{i = 1}^{n} {\delta_{i}^{2} - \sum\limits_{i = 1}^{n} {\alpha_{i} } \left\{ {\left\langle {w,\Phi \left( {\varvec{X}} \right)} \right\rangle + b + \delta_{i} - Y_{i} } \right\}}$$

can be obtained by partial differentiation for $w$, $b$, $\delta$ and $\alpha$, respectively:

16 $$\left\{ {\begin{array}{*{20}l} {\frac{\partial L}{{\partial w}} = 0 \to w = \sum\limits_{i = 1}^{n} {\alpha_{i} \Phi ({\varvec{X}}_{i} )} } \hfill \\ {\frac{\partial L}{{\partial b}} = 0 \to \sum\limits_{i = 1}^{N} {\alpha_{i} = 0} } \hfill \\ {\frac{\partial L}{{\partial \delta }} = 0 \to \alpha_{i} = C\delta_{i} } \hfill \\ {\frac{\partial L}{{\partial \alpha_{i} }} = 0 \to \left\langle {{\varvec{w}},\Phi \left( {{\varvec{X}}_{i} } \right)} \right\rangle + b + \delta_{i} - Y_{i} = 0} \hfill \\ \end{array} } \right.$$

17 $$\left[ {\begin{array}{*{20}l} 0 & {{\varvec{E}}^{T} } \\ {\varvec{E}} & {{\varvec{ZZ}}^{T} + C^{ - 1} {\varvec{I}}} \\ \end{array} } \right]\left[ {\begin{array}{*{20}c} b \\ {\varvec{A}} \\ \end{array} } \right] = \left[ {\begin{array}{*{20}c} 0 \\ {\varvec{Y}} \\ \end{array} } \right]$$

where $E = (1,1, \cdots ,1)^{T}$,$Z = \left[ {\phi (X_{1} ),\phi (X_{2} ), \cdots ,\phi (X_{n} )} \right]^{T}$, $A = (\alpha_{1} ,\alpha_{2} , \cdots \alpha_{n} )^{T}$,$Y = (y_{1} ,y_{2} , \cdots y_{n} )^{T}$, and $I$ is the unit matrix. The kernel function $K(x,x_{i} ) \le \phi (x)$, $\phi (x_{i} ) >$ is chosen to reduce the computational effort, then the regression equation of the LSSVM model is finally determined as:

18 $$f(X) = \sum\limits_{i = 1}^{n} {\alpha_{i} K({\varvec{X}},{\varvec{X}}_{i} ) + b}$$

## Monthly precipitation prediction model

### Prediction steps

The construction steps of the monthly precipitation prediction model based on the AVOA and VMD are outlined as follows:

*Step 1*: Utilize the VMD algorithm to decompose the precipitation sequence based on different stepwise decomposition sample construction methods, resulting in K modal components.

*Step 2*: To precisely describe the preceding influencing factors for each component, determine the corresponding lag months (lag_k_) for the kth modal component based on AutoCorrelation Function and Partial AutoCorrelation Function. Taking Huairou Station as an example, six modal components are obtained with lag values of [2, 7, 4, 6, 6, 3] for each component.

*Step 3*: Generate training and testing samples based on different stepwise decomposition sample construction methods, with a training sample ratio of 0.8 and the remaining as testing samples. Normalize the samples according to the training set.

*Step 4*: The training samples are fed into the prediction model for training.

**Figure 1:**
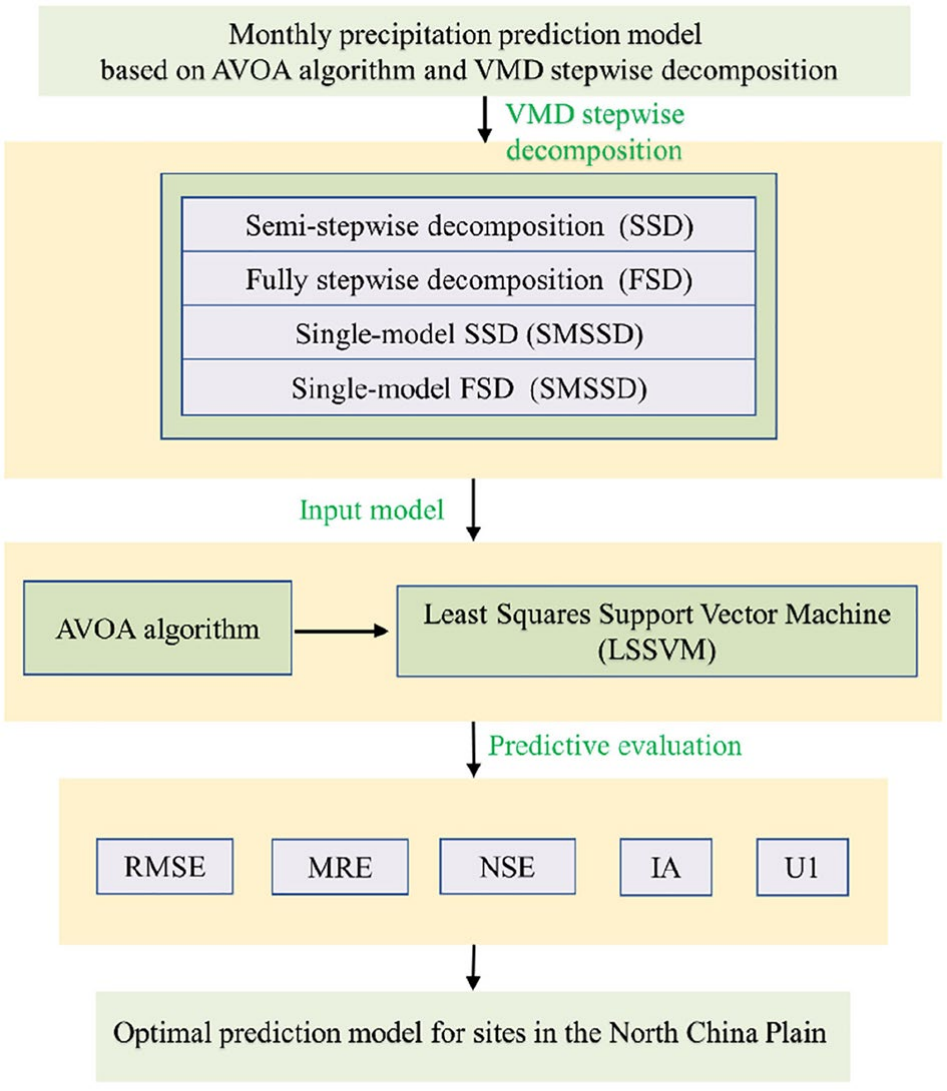
Construction steps of the monthly precipitation prediction model based on AVOA algorithm and VMD stepwise decomposition.

*Step 5*: The accuracy and performance of the model is evaluated through evaluation metrics. The flowchart of the monthly precipitation prediction model is shown in Fig. 1.

### Evaluation metrics

Model error evaluation indexes include the root mean square error (RMSE), Nash efficiency coefficient (NSE), and mean relative error (MRE). The lower the error, the higher the prediction accuracy; the consistency index (IA), which ranges from 0 to 1, reflects the generalization ability; the closer it is to 1, the better the model prediction performance; and the U-statistic (U1), which evaluates the prediction ability, the closer it is to 0. The model's predictive power increases with the value's proximity to zero. The following is the calculating formula:

19 $$RMSE = \sqrt {\frac{{\sum\limits_{i = 1}^{n} {(P\left( i \right) - P_{{}}^{*} \left( i \right))^{2} } }}{n}}$$

20 $$NSE = 1 - \frac{{\sum\limits_{i = 1}^{n} {\left( {P\left( i \right) - P_{{}}^{*} \left( i \right)} \right)^{2} } }}{{\sum\limits_{i = 1}^{n} {\left( {P\left( i \right) - \overline{P} } \right)^{2} } }}$$

21 $$MRE = \left( {\frac{1}{n}\sum\limits_{i = 1}^{n} {\left| {\frac{{P\left( i \right) - P_{{}}^{*} \left( i \right)}}{P\left( i \right)}} \right|} } \right) \times 100\%$$

22 $$IA = 1 - \frac{{\sum\limits_{i = 1}^{n} {\left( {P\left( i \right) - P^{*} \left( i \right)} \right)}^{2} }}{{\sum\limits_{i = 1}^{n} {\left( {\left| {P\left( i \right) - \overline{P}\left( i \right)} \right| - \left| {P^{*} \left( i \right) - \overline{P}\left( i \right)} \right|} \right)}^{2} }}$$

23 $$U1 = \frac{{\sqrt {\frac{1}{n}\sum\limits_{i = 1}^{n} {\left( {P\left( i \right) - P^{*} \left( i \right)} \right)}^{2} } }}{{\sqrt {\frac{1}{n}\sum\limits_{i = 1}^{n} {P\left( i \right)}^{2} } + \sqrt {\frac{1}{n}\sum\limits_{i = 1}^{n} {P^{*} \left( i \right)}^{2} } }}$$

In these formulas, *P* represents the actual values, P* denotes the model-predicted values, and $\overline{P}$ is the mean of the actual value sequence.

## Case analysis

### Data source

The North China Plain covers a total area of 300,000 km^2^, belonging to the continental monsoon climate zone, with obvious changes in the four seasons and an average annual precipitation of 500–900 mm. Alluvial plains are characterized by comparatively flat topography, with the majority of elevations being below 50 m^28^. One of China's most significant bases for grain production, the North China Plain is crucial to the country's food security. Making timely and efficient judgments about agricultural productivity and water resource management can be aided by predictive rainfall simulation in this area^29^.

**Figure 2:**
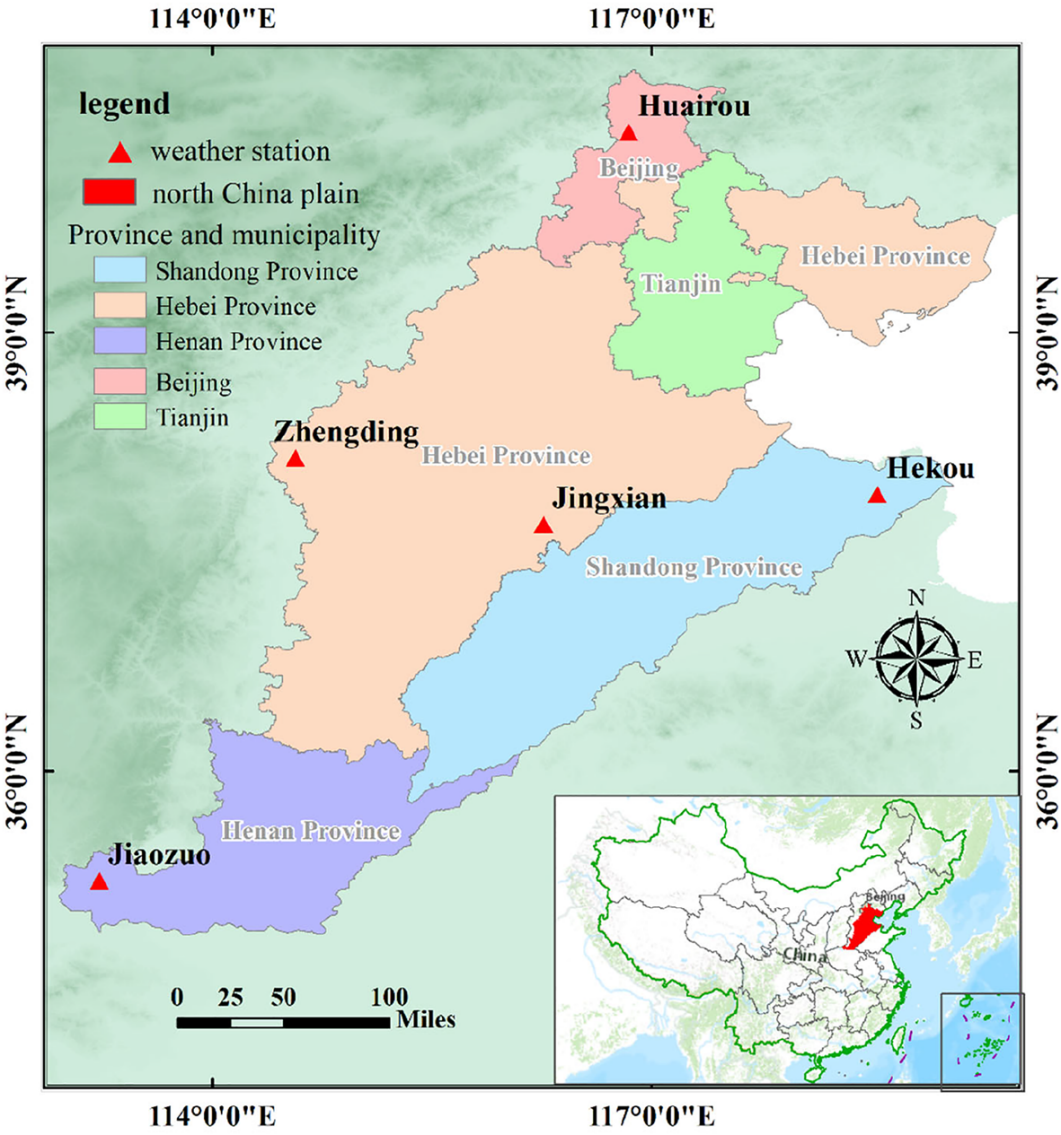
Distribution map of the study sites.

Given the climatic variations across the North China Plain, the study focuses on five meteorological stations as research objects: Huairou (116°38′E, 40°22′N), Zhengding (114°34′E, 38°9′N), Jingxian (116°17′E, 37°42′N), Hekou (118°32′E, 37°53′N), and Jiaozuo (113°16′E, 35°14′N). The distribution of these locations is illustrated in Fig. 2. The map in Fig. 2 was created using the ArcGIS software version10.8, available at http://www.esri.com/software/arcgis.

The North China Plain's national meteorological stations provided the precipitation data used in the meteorological analysis (https://data.cma.cn/). For the estuary stations, precipitation data spanning January 1993 to December 2018 is available. Precipitation data from January 1973 to December 2018 are available for the remaining locations.

### Sample construction

The ratio of training and testing samples for the model was taken as 4:1. The training period was from August 1972 to July 2009 and the testing period was from August 2009 to December 2018, except for the estuary station (training period: July 1992 to July 2013; testing period: August 2013 to December 2018).

Constructing correct and effective training and test samples is the focus of accurate prediction of precipitation. Commonly employed sample construction methods conducive to practical application include the "Semi-stepwise Decomposition (SSD)" sample technique^30^, the "Fully Stepwise Decomposition (FSD)" sample technique^31^, the "Single-model Semi-stepwise Decomposition (SMSSD)" sample technique^32^, and the "Single-model Fully Stepwise Decomposition (SMFSD)" sample technique^20^. The first two methods require the simultaneous construction of K (where K represents the number of modal components) models. In contrast, the latter two methods only necessitate a single model to obtain the final prediction result, thereby offering a higher operational speed.

**Figure 3:**
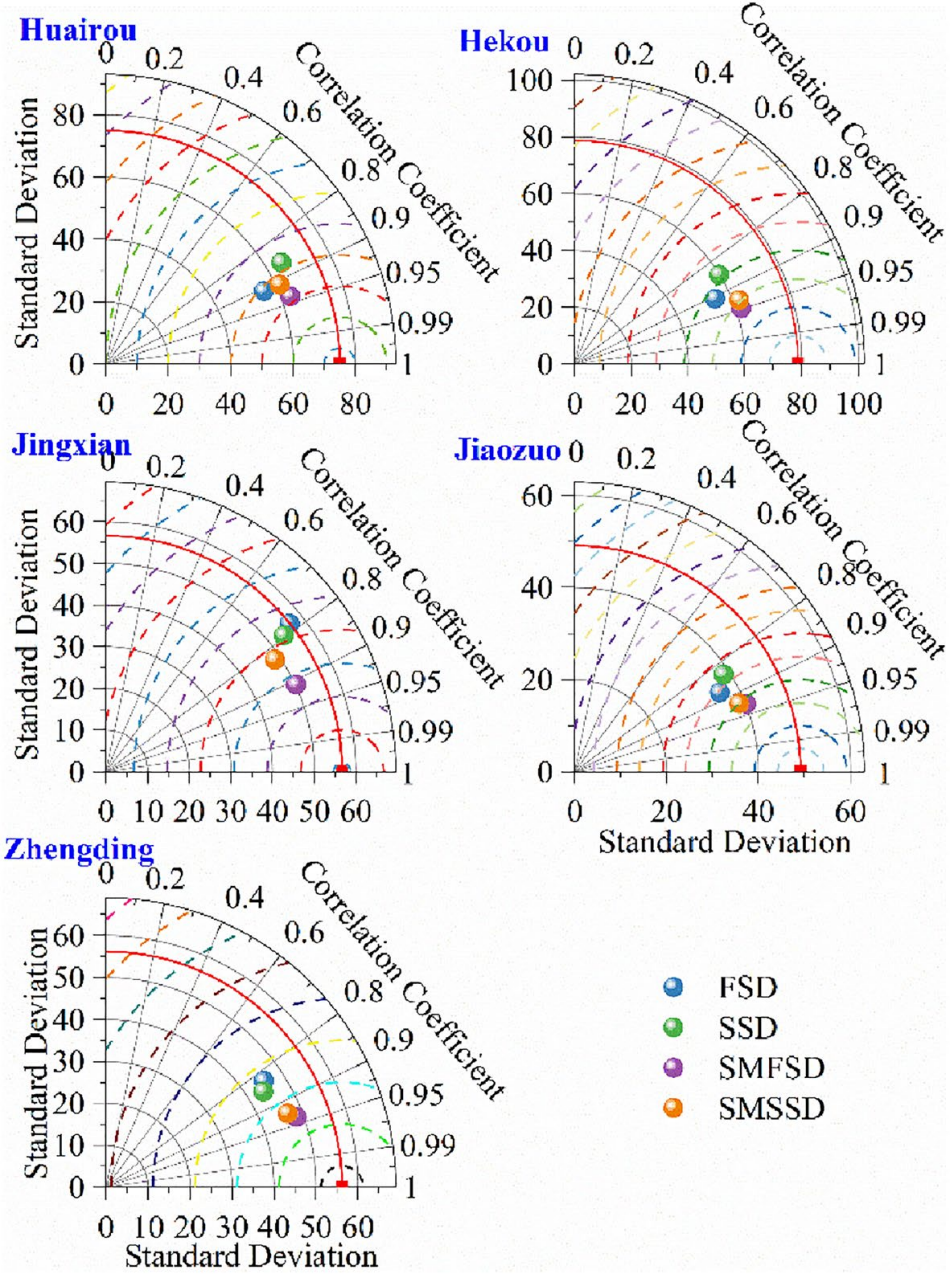
Taylor distribution for different sampling methods.

Using VMD and the four sampling strategies, the rainfall data were broken down into subsequences, which were then fed into the LSSVM model to be simulated. Based on rainfall data from five locations in the North China Plain, a comparative analysis was carried out. As seen in Fig. 3, the radial line depicts the correlation coefficient, the horizontal and vertical axes reflect the standard deviation, and the scattered spots in the picture represent various sampling techniques. It is evident that the five stations perform better when using the stepwise decomposition approaches (SMFSD and SMSSD), and the predicted outcomes of these sampling strategies are most similar to the observed values with the least amount of standard deviation. Among the four sample approaches, the majority of correlation coefficients fall between 0.8 and 0.95.

### Suitability of parameter α

The penalty factor α, as a crucial parameter for precipitation sequence denoising, plays an equally significant role in forecasting accuracy. Three scenarios with penalty factor α set to 100, 1000, and 2000 were considered. Since only the testing period results can reflect actual predictive capabilities, Friedman tests were conducted for a total of 12 composite models, encompassing the three α values. The results, as presented in Table 1, indicate that the average ranks for the models corresponding to α values 100, 1000, and 2000 are 13.83, 12.70, and 11.55, respectively. Larger α values within the 2000 range are more conducive to precipitation forecasting.

**Table 1:** Friedman test results for different α values.

Models	α = 100		α = 1000		α = 2000	
	Average Ranks	Ranking	Average Ranks	Ranking	Average Ranks	Ranking
FSD-AVOA-LSSVM	16.2	7	10.8	6	9.67	5
SMFSD-AVOA-LSSVM	9.4	4	5.87	**2**	4.93	**1**
SMSSD-AVOA-LSSVM	16.47	10	16.53	11	15.87	9
SSD-AVOA-LSSVM	20.2	13	17.6	12	15.73	8
AVOA-LSSVM	6.87	3	–	–	–	–
Mean	13.83		12.7	–	11.55	–

Significant values are in bold.

The composite model based on SMFSD technique consistently maintains its top two positions, continuing to uphold its superiority over other decomposition techniques. In contrast, the AVOA-LSSVM model ranks third. Therefore, the utilization of inappropriate decomposition techniques in monthly precipitation forecasting may result in decreased accuracy. The SMFSD technique emerges as a more suitable sample construction method for predicting monthly precipitation in the North China Plain.

### Suitability across different stations

The above study focuses on the overall evaluation of the performance of stepwise decomposition techniques in the North China Plain. However, due to the vast geographical range leading to differences in geographical and climatic conditions, the adaptability of each model may vary across different stations. The average predictive success rate of each model is used to determine the optimal applicable model for each station. According to the Hydrological Information Forecast Specification (GB/T 22482-2008), the permissible error is determined based on a 20% range of the measured values during the same period over multiple years.

**Figure 4:**
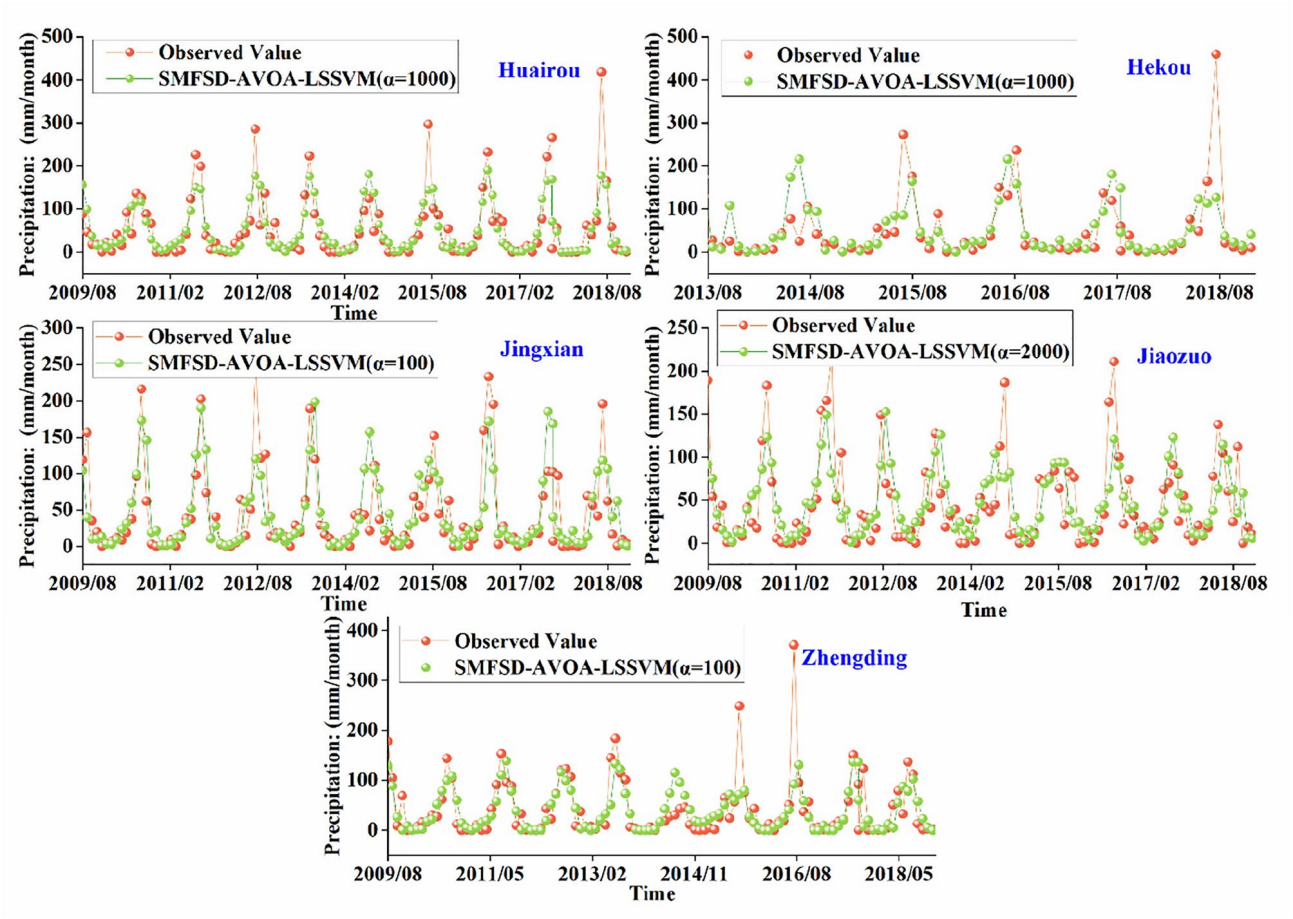
Optimal prediction results for each station in the North China Plain.

According to this requirement, the average success rate of each model at each station is calculated for each month. Similarly, Friedman tests are used to rank the models. Table 2 records the optimal models for each station, along with their success rates in each month and the p-values from the Friedman test. Based on a confidence level of 0.05 and the p-values, the model ranking results are only significantly different for Huairou and Zhengding stations. The optimal monthly precipitation prediction model for Zhengding station is SMFSD-AVOA-LSSVM with α = 100, while for Huairou station, it is SMFSD-AVOA-LSSVM with α = 1000. Using an 80% threshold as the preferred criterion to determine the advantageous months for each station in prediction work, the results are as follows: June for Huairou station (87.8%), February (90.0%) and October (86.7%) for Hekou station, July for Jiaozuo station (88.9%), May (88.9%) and August (90.0%) for Zhengding station. At the Jingxian station, the months of May (76.7%) and September (79.0%) are closer to the preferred threshold. In terms of the average success rate, only Zhengding station exceeds 60%. The average success rate at Hekou station is the lowest, only 48.8%, possibly due to insufficient learning caused by limited historical data. Table 2 provides the test results for the optimal models at each station, and Fig. 4 illustrates the training and prediction effects at each station. The best predictive performance in this study is observed at Huairou and Jingxian stations, while Hekou station exhibits the poorest predictive performance. Additionally, the prediction of precipitation sequences at each station performs well in the low-value range, while the predictive ability near extreme values requires further.

**Table 2:** Optimal models for each station based on monthly success rate.

Station	Huairou	Hekou	Jingxian	Jiaozuo	Zhengding
Optimal model	SMFSD-AVOA-LSSVM (α = 1000)	SMFSD-AVOA-LSSVM (α = 1000)	SMFSD-AVOA-LSSVM (α = 100)	SMFSD-AVOA-LSSVM (α = 2000)	SMFSD-AVOA-LSSVM (α = 100)
*P* value	5.71E−05	5.98E−01	4.78E−01	9.93E−01	3.58E−02
Success rate (%)					
Jan	72.2	64.0	72.2	44.4	66.7
Feb	58.9	90.0	65.6	58.9	77.8
Mar	45.6	16.0	36.7	46.7	66.7
Apr	60.0	38.0	44.4	55.6	63.3
May	60.0	58.0	76.7	65.6	88.9
Jun	87.8	8.0	57.8	73.3	55.6
Jul	66.7	28.0	50.0	88.9	77.8
Aug	65.0	45.0	75.0	60.0	90.0
Sept	66.0	25.0	26.0	42.0	54.0
Oct	65.0	86.7	79.0	40.0	12.0
Nov	40.0	61.7	61.0	22.0	32.0
Dec	30.0	65.0	36.0	28.0	50.0
Average value (%)	59.8	48.8	56.7	52.1	61.2

### Comparison of simulation results

While Fig. 4 shows the comparison between the prediction effect and the actual rainfall during the test period at each site, Table 3 provides the findings of the evaluation indexes of the ideal models at each site. Based on the error indicator results used to assess the model's prediction accuracy, the NSE ranges from 0.73 to 0.86, with Huairou and Jingxian having the best NSE; the RMSE of SMFSD-AVOA-LSSVM is less than 25 mm, with the lowest value being 11.62 mm in Jiaozuo. The IA results show that generalization ability is a key metric for assessing model prediction accuracy; the closer the generalization ability is to 1, the better. All five of the North China Plain stations have IAs better than 0.96, demonstrating the good prediction performance and good generalization capacity of SMFSD-AVOA-LSSVM. U1 is used to measure the prediction abilities of the model; the closer the model is to 0, the better. The three with the finest prediction skill and the least U1 scores are Huairou, Jingxian, and Zhengding. In this study, Huairou and Jingxian stations have the best prediction performance when combining the least error, optimal generalization ability, and prediction ability.

**Table 3:** Results of optimal model evaluation indicators for each site.

Indicators	Huairou	Hekou	Jingxian	Jiaozuo	Zhengding
RMSE	18.37	25.11	24.74	11.62	14.32
NSE	0.86	0.75	0.86	0.80	0.79
MRE	107.20	173.75	51.71	106.26	96.17
IA	0.97	0.96	0.98	0.97	0.98
U1	0.11	0.15	0.11	0.12	0.11

As shown in Fig. 4, the predicted precipitation series curves for each station are better predicted in the low value range, while the prediction ability near the extremes needs to be strengthened.

## Conclusion

The ability of SSD, FSD, SMSSD, and SMFSD to provide support for actual monthly precipitation forecasts was further examined through the combined AVOA-LSSVM model.

1. Considering the differences in sample construction techniques and the impact of different VMD parameters α, the optimal model identified is the SMFSD-AVOA-LSSVM with α = 1000, suitable for Huairou and Jingxian stations, with an average success rate of 59.8% and 56.7%, respectively. The model suitable for the monthly precipitation forecast at the Zengding station is the SMFSD-AVOA-LSSVM with α = 100, achieving an average success rate of 61.2%. For the Hekou station, the model best suited is the SMFSD-AVOA-LSSVM with α = 1000, yielding an average success rate of 48.8%. The model suitable for the monthly precipitation forecast at the Jiaozuo station is the SMFSD-AVOA-LSSVM with α = 2000, resulting in an average success rate of 52.1%.
2. The SMFSD sample technique stands out as the most suitable tool for monthly precipitation forecasting in the North China Plain. Combining error and validation assessment results, the overall best predictive performance is observed at Huairou station (RMSE of 18.37mm, NSE of 0.86, MRE of 107.2%, IA = 0.97, U1 = 0.11) and Jingxian station (RMSE of 24.74mm, NSE of 0.86, MRE of 51.71%, IA = 0.98, U1 = 0.11), while Hekou station exhibits the poorest overall performance (RMSE of 25.11mm, NSE of 0.75, MRE of 173.75%, IA = 0.96, U1 = 0.15).
3. SMFSD uses a single model in each step of the decomposition for a comprehensive decomposition. This approach ensures that global information is preserved at each step, allowing consistency and completeness of data features across all steps. This avoids loss of information and improves the overall performance of the forecasting model. SMFSD combines the African Vulture Optimisation Algorithm (AVOA) and Least Squares Support Vector Machines (LSSVM) to optimise the model parameters at each decomposition step, allowing the model to better fit the time series data.

The focus of this study is on rainfall prediction in the North China Plain, which may limit the applicability of the results to other regions with different climatic characteristics. Although the study evaluated model performance using metrics such as RMSE, NSE, IA and U1, it may lack a comprehensive assessment of forecast uncertainty, robustness under extreme conditions or comparison with other modelling approaches. This paper only compares the variability of one decomposition model, VMD, across different sampling approaches, and does not compare other decomposition models, which is methodologically deficient, and should be further explored in the future for the differences between different decomposition models in terms of stepwise decomposition sampling approaches.

## Data Availability

Data and materials are available from the corresponding author upon request.
